# Silent Gastroesophageal Reflux Disease in Patients with Morbid Obesity Prior to Primary Metabolic Surgery

**DOI:** 10.1007/s11695-020-04959-6

**Published:** 2020-09-10

**Authors:** Ivan Kristo, Matthias Paireder, Gerd Jomrich, Daniel M. Felsenreich, Mario Fischer, Florian P. Hennerbichler, Felix B. Langer, Gerhard Prager, Sebastian F. Schoppmann

**Affiliations:** 1 Department of Surgery, Upper GI Research & Service, Medical University of Vienna, Währinger Gürtel 18-20, 1090 Vienna, Austria; 2 Department of Surgery, Metabolic & Bariatric Surgery, Medical University of Vienna, Vienna, Austria

**Keywords:** GERD, Morbid obesity, Asymptomatic, Lyon, Manometry, pH metry

## Abstract

**Purpose:**

Long-term follow-up after sleeve gastrectomy (SG) revealed a high incidence of gastroesophageal reflux disease (GERD) frequently caused by preoperative silent pathologic reflux. We aimed to evaluate prevalence and phenotypes of GERD in asymptomatic patients with morbid obesity prior to metabolic surgery according to modern objective testing.

**Material and Methods:**

Prospective collection of data including consecutive patients with morbid obesity (body mass index (BMI) ≥ 35 kg/m^2^) prior to metabolic surgery was applied for this study between 2014 and 2019. Patients underwent clinical examinations, endoscopy, pH metry, and high-resolution manometry and were analyzed according to the Lyon consensus.

**Results:**

Of 1379 patients undergoing metabolic surgery, 177 (12.8%, females = 105) asymptomatic individuals with a median age of 42.6 (33.8; 51.6) years and a median BMI of 44.6 (41.3; 50.8) kg/m^2^ completed objective testing and were included during the study period. GERD was diagnosed in 55 (31.1%), whereas criteria of borderline GERD were met in another 78 (44.1%). GERD was mediated by a structural defective lower esophageal sphincter (*p* = 0.004) and highlighted by acidic (*p* = 0.004) and non-acidic (*p* = 0.022) reflux episodes. Esophageal motility disorders were diagnosed in 35.6% (*n* = 63) of individuals with a novel hypercontractile disorder found in 7.9% (*n* = 14) of patients.

**Conclusion:**

GERD affects a majority of asymptomatic patients with morbid obesity prior to primary bariatric surgery. Future longitudinal trials will have to reveal the clinical significance of esophageal motility disorders in patients with morbid obesity.

## Introduction

Adverse life style behavior and obesity represent demanding challenges in modern medicine. By 2030, 1 in 2 adults is projected to have obesity, whereas 1 in 4 adults will even have severe obesity, which is likely to become the most common body mass index (BMI) category among women [1]. As a consequence, related chronic diseases will affect general health status and increase the socioeconomic burden [2].

As far as weight loss and remission rates of type II diabetes and metabolic syndrome are concerned, metabolic surgery is more effective when compared with non-surgical options and was therefore implemented as an effective strategy fighting obesity [3].

Currently, various primary operations are performed throughout the world with sleeve gastrectomy (SG) being the predominant procedure [4]. Importantly, long-term follow-up after SG revealed a high incidence of gastroesophageal reflux disease (GERD) and Barrett’s esophagus, a premalignant condition caused by reflux of gastric contents [5]. Undetected preoperative silent GERD may attribute to these outcomes [6]. Thus, SG is controversial in patients with pre-existing pathologic reflux.

However, diagnosis of GERD prior to metabolic surgery is more than challenging as symptoms are misleading or even missing on the one side, whereas esophagitis, detected by endoscopic means, may also be present in healthy controls on the other side [7, 8]. As a consequence, GERD may remain undiscovered in asymptomatic individuals.

The Lyon consensus, a novel approach that integrates objective tools to modernize diagnosis of GERD, differentiates between no, borderline, or conclusive evidence for pathologic reflux [9]. Recently, first data applying the Lyon consensus revealed a high prevalence of GERD and motility disorders in the population with morbid obesity [10]. Unfortunately, data of asymptomatic patients with morbid obesity classified by the Lyon consensus have yet to be published.

This study was designed to evaluate the prevalence and phenotypes of GERD and motility disorders in a large cohort of asymptomatic patients with morbid obesity prior to primary metabolic surgery.

## Material and Methods

Consecutive asymptomatic patients with morbid obesity (BMI ≥ 35 kg/m^2^), planned for primary metabolic surgery, were included in this study between 2014 and 2019 at our tertiary academic center. Patients were assigned for objective testing for GERD according to the Lyon consensus including ambulatory 24-h pH impedance monitoring and high-resolution manometry (HRM). Esophagogastroduodenoscopy (EGD) was performed to determine presence of hiatal hernia and esophagitis, classified according to the Los Angeles (LA) classification [11]. Hiatal hernia was diagnosed when greater than 2 cm in axial span. Small hiatal hernias were measured between 2 and 4 cm, whereas large hiatal hernias were considered to be larger than 4 cm. Medical history as well as baseline characteristics and a validated GERD-health–related quality of life questionnaire were assessed in face-to-face interviews at the day of esophageal function testing following a standardized documentation [12]. Patients were declared asymptomatic in case of absence of typical and atypical GERD-related symptoms and a scoreless GERD-HRQL [9]. All participants were free of anti-secretory medication during objective testing. Only patients with completed objective testing were included and presented in this trial. Esophageal function testing is routine practice for patients undergoing evaluation for anti-reflux surgery at our institution. The local institutional review board approved the study protocol and informed consent was obtained from all patients. Data were collected prospectively.

### Ambulatory 24-H pH Impedance Monitoring

A catheter containing impedance tracers and electrodes with internal reference for pH measuring was utilized (ComforTec ZAN-44; Sandhill Scientific, Highlands Ranch, CO, USA). Electrodes were positioned after locating the lower esophageal sphincter (LES) by HRM. Patients were instructed to stick to their daily routine and specify body position and meals. GERD was defined following the Lyon consensus statement as the percentage of endoluminal pH < 4 exceeding 6% in the distal esophagus and/or endoscopic visible lesions grade C or D according to the LA classification [9]. Diagnosis of borderline GERD included patients with a total acid exposure time between 4 and 6%, presence of grade A and B esophagitis, and reflux episodes exceeding 40 within 24 h.

### High-Resolution Impedance Manometry

HRM was performed using solid-state catheters with esophageal body motility being assessed with 10 liquid swallows of 5 ml at 30-s intervals. Data were interpreted according to the Chicago classification 3.0 [13]. A total length below 2 cm and/or an intraabdominal length lower than 1 cm defined a structurally defective LES. The LES was considered hypotensive below 10 mmHg and hypertensive when exceeding 45 mmHg.

### Statistical Analysis

Demographics are presented as mean with standard deviation if normally distributed, or otherwise as median with interquartile ranges. Categorical variables are displayed as absolute numbers and percentages. HRM metrics are delineated as median with interquartile ranges. Chi-square or Wilcoxon rank test was applied as appropriate for comparison between groups. *p* values ≤ 0.05 were considered statistically significant. Analyses were done using SPSS for Macintosh Version 24.0 (IBM Corp., Armonk, NY, USA).

## Results

### Baseline Data

**Fig. 1:**
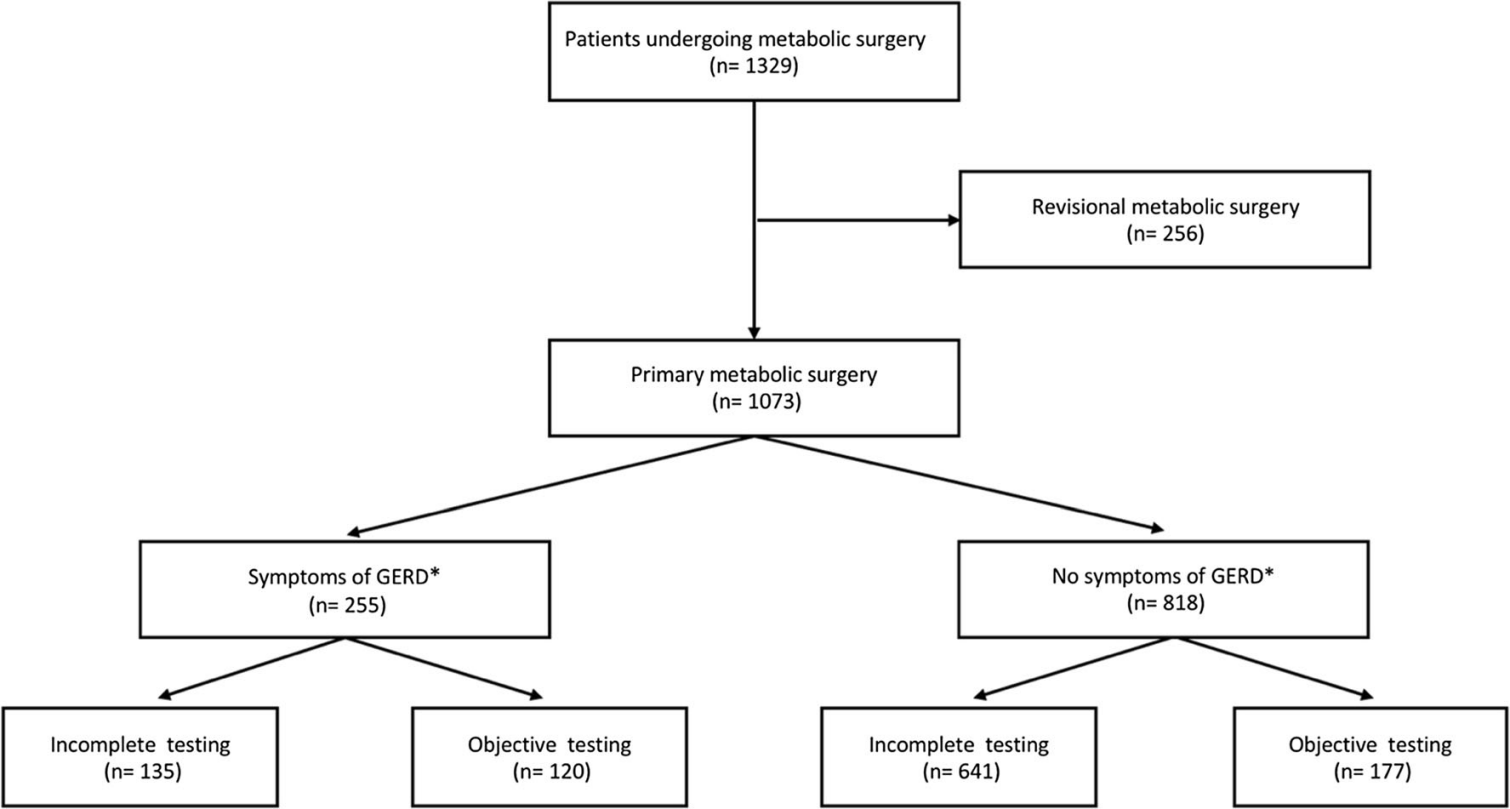
Flow of patients from undergoing metabolic surgery to complete objective testing for gastroesophageal reflux disease between 2014 and 2019. *Gastroesophageal reflux disease

Of 1379 individuals undergoing metabolic surgery during the study period, 177 (12.8%) patients (females = 105, 59.3%) with a median age of 42.6 (33.8; 51.6) years and a median BMI of 44.6 (41.3; 50.8) kg/m^2^ presented asymptomatic and completed full objective testing during the study period (Fig. 1). Further characteristics are outlined in Table 1.

**Table 1:** Baseline characteristics of asymptomatic morbidly obese patients undergoing primary bariatric surgery

Patients	177
Sex, *n* (%)	
Female	105 (59.3)
Male	72 (40.7)
Waist-to-hip ratio	1.0 (±0.7)
Diabetes	46 (26.0)
Habits, *n* (%)	
Smoking	66 (37.2)
Alcohol consumption	
Daily	1 (0.7)
Frequently	58 (32.8)
Never	118 (66.7)
Endoscopy, *n* (%)	
Hiatal hernia	57 (32.2)
Esophagitis	46 (26.0)

### Asymptomatic GERD

Analysis of data revealed evidence of GERD in 55 (31.1%) asymptomatic patients, whereas criteria of borderline GERD were met in 78 (44.1%). EGD revealed esophagitis in 46 (26%) participants and presence of hiatal hernia in 57 (32.2%). Grade A esophagitis was observed in 19 (10.7%) patients, whereas grades B, C, and D were noted in 18 (10.2%), 6 (3.4%), and 3 (1.7%), respectively. Considering hiatal hernias, 46 (26%) patients were measured to have small and 11 (6.2%) to have large hiatal hernias. Individuals with hiatal hernia were more likely to be diagnosed with esophagitis (*p* = 0.003) but only showed a trend towards GERD (*p* = 0.054). Patients with GERD had a shorter total LES length (*p* = 0.004) and a shorter intraabdominal LES fraction (*p* = 0.002) noted during manometric evaluation. During pH impedance monitoring, GERD was highlighted by an increased number of acidic (*p* = 0.004) and non-acidic (*p* = 0.022) reflux episodes. There were no other baseline parameters associated with the diagnosis of GERD. Full data are presented in Table 2.

**Table 2:** Comparison of asymptomatic morbidly obese patients prior to bariatric surgery with/without evidence of (borderline) GERD

	(Borderline) GERD	No GERD	*p* value
Patients	133	44	
Sex, *n* (%)			NS
Female	75 (42.4)	30 (16.9)	
Male	58 (32.8)	14 (7.9)	
Age	42.8 (± 12.0)	44.4 (± 12.9)	NS
BMI	46.4 (± 6.7)	47.4 (± 8.3)	NS
Waist-to-hip ratio	1.02 (± 0.8)	0.95 (± 0.1)	NS
Diabetes	32 (18.1%)	14 (7.9%)	NS
Habits, *n* (%)			
Smoking	49 (27.7)	17 (9.6)	NS
Alcohol consumption			NS
Daily	1 (0.6)	0	
Frequently	46 (26.0)	12 (6.8)	
Never	85 (48.0)	32 (18.1)	
Endoscopy, *n* (%)			
Hiatal hernia	48 (27.1)	9 (5.1)	0.05
Esophagitis	45 (25.4)	1 (0.6)	0.0001
High-resolution manometry, *n* (%)			
Esophageal motility disorder	47 (26.6)	16 (9.0)	NS
Lower esophageal sphincter			
Structurally defective	52 (29.4)	12 (6.8)	NS
Short total length	36 (20.3)	9 (5.1)	NS
Short intraabdominal fraction	49 (27.7)	8 (4.5)	0.02
Hypertensive	22 (12.4)	12 (6.8)	NS
Hypotensive	10 (5.6)	0	NS
Complete bolus rate (%)	72 (± 40)	76 (± 36)	NS
Impedance pH metry			
Time pH < 4 (%)			
Total	4.6 (2.6; 7.6)	1.1 (0.7; 2.1)	0.0001
Recumbent	0.1 (1.4; 4.6)	0 (0; 0.3)	0.0001
Upright	6 (2.9; 9.2)	1.6 (1.1; 3.6)	0.0001
Reflux episodes			
Acidic	33 (24; 45)	13 (5; 23)	0.0001
Non-acidic	15 (8; 23)	12 (7; 17)	0.05
Gas	4 (1; 10)	5 (3; 10)	NS

### High-Resolution Manometry

HRM detected a structurally defective LES in 64 (36.2%) patients with a decreased total length and intraabdominal fraction observed in 45 (25.4%) and 57 (32.2%) individuals, respectively. Hypertensive LES was observed in 34 (19.2%) study participants, whereas hypotensive LES was registered in 10 (5.6%).

The Chicago classification revealed motility disorders in 63 (35.6%) patients. Esophagogastric junction outflow obstruction (EGJOO) was present in 31 (17.5%), Jackhammer esophagus (JE) in 14 (7.9%), distal esophageal spasm (DES) in 10 (5.6%), ineffective esophageal motility (IEM) in 6 (3.4%), fragmented peristalsis in 1 (0.6%), and type III achalasia in 1 (0.6%) patients. Concomitant outflow obstruction was noted in 64.3% (*n* = 9) of patients with JE and 60% (*n* = 6) of individuals with distal esophageal spasm.

## Discussion

This is the first study investigating a large sample of asymptomatic patients with morbid obesity prior to primary metabolic surgery following a modern concept of GERD. According to the Lyon consensus, 31.1% of our patients were diagnosed with GERD, whereas another 44.1% fulfilled the criteria for borderline GERD. Reflux was facilitated by a structurally defective sphincter. Furthermore, esophageal motility disorders, mainly presenting as novel spastic or obstructive phenotypes, were observed in 35.6% of asymptomatic individuals.

Metabolic surgery and the development of postoperative, even complicated, GERD are intensively discussed in literature. Currently, SG is the most frequently performed metabolic procedure worldwide but was recently associated with some limitations concerning GERD [4]. We investigated our first SG patients and noted a high rate of postoperative GERD and de novo hiatal hernia 10 years after SG [5]. Pathologic reflux was observed in 57% of the unconverted cohort and even a rate of 14% of Barrett’s metaplasia, a premalignant condition. Furthermore, symptomatic GERD was one of the major reasons for conversion in this study. Confirmatory, these findings were reproduced in other centers and significantly impact on quality of life after metabolic surgery [14]. From a pathophysiological point of view, Quero et al. revealed several mechanisms that may facilitate pathologic reflux after SG [15]. They evaluated 23 patients by means of magnetic resonance imaging and HRM before and after surgery and realized that LES length was reduced after SG, aggravating our findings, where even 36.2% of asymptomatic patients had a structurally defective LES prior to surgery. Moreover, disruption of the esophagogastric junction and small gastric capacity were associated with the risk of postoperative GERD leading to concepts of novel sphincter augmentation devices being implemented for these patients [16]. Therefore, it seems reasonable that preoperative assessment of LES function could help us tailor our surgical decision-making to prevent additional damage to an already injured sphincter. Although literature is controversial on this issue, it seems that structural defects play an important role in the pathogenesis of GERD [17].

As a consequence, SG may be questioned in individuals with pre-existing sphincter defects or evidence of GERD, which confronts us with several limitations [18]. First and foremost, GERD patients may present asymptomatic like our selected population. Furthermore, silent preoperative reflux seems to play a crucial role in the development of postoperative GERD. Borbély et al. evaluated 116 patients with symptoms of GERD after SG and reported that 66% of patients with preoperative silent GERD became symptomatic after interventions [6]. Furthermore, preoperative objective testing for GERD and LES assessment is not mandatory, which leads to a symptom- or endoscopic-based diagnosis of GERD. Unfortunately, the Diamond trial revealed that symptom-based diagnosis of GERD lacks sensitivity and specificity, even in an expert setting, where it does not exceed 70% [19]. Moreover, esophagitis was observed in up to 11.2% of young healthy volunteers during endoscopy [20]. Due to these issues, the Lyon consensus established objective elements of reflux testing to define borders of abnormality. Importantly, according to the Lyon consensus, 31.1% of our population were diagnosed with GERD, whereas another 44.1% had (borderline) GERD. This results in 75.2% of asymptomatic individuals with morbid obesity being affected by some form of GERD prior to metabolic surgery. This is difficult to compare as objective data prior to metabolic surgery are rare. There is only one trial that identifies patients with GERD using the Lyon consensus in the predominantly symptomatic population with morbid obesity [10]. Within this study, 35.4% of individuals presented with GERD and another 40.8% with (borderline) GERD, which is comparable to our data. Nevertheless, we have to point out that we only included asymptomatic patients, which again highlights the controversial issue of symptom and endoscopic-orientated selection of surgical strategy. Lower quality data, biased by the limitations of endoscopic diagnosis without histopathology of GERD, report prevalence rates of silent GERD starting from 11.6% in the general population [21]. But again, the presence of low-grade esophagitis may be misleading.

Although variations of BMI did not influence the presence of GERD in our cohort of morbidly obese, several facts explain the high prevalence of GERD. Obesity has been established as an important risk factor for occurrence of GERD but central obesity seems to be a better prediction marker when compared with BMI [22, 23]. High pressure gradients registered in the population with morbid obesity explain a hiatal hernia rate of 32.2% and facilitate reflux via a defective sphincter in 36.2% of our participants. Additionally, obesity may lead to central and peripheral neuronal damage and increase the sensory threshold leading to diminished symptom perception [24].

Importantly, high-resolution manometry modernized our understanding of esophageal disorders. Our group noted motility disorders in 35.6% of study participants which is similar to a recent study in the general population of morbid obese prior to bariatric surgery [10]. Interestingly, JE, a novel hypercontractile disorder noted in 7.9% of asymptomatic individuals within this trial, is defined by the Chicago classification not to be present in controls and represents a clear hypercontractile abnormality. Recently, JE was associated with a progressive clinical nature with 25% of individuals even developing achalasia in short-term follow-up [25]. Therefore, aggressive treatment with tailored peroral endoscopic myotomy (POEM) is offered to individuals with JE [26]. However, the underlying mechanism in the asymptomatic morbidly obese and therapeutic consequences are not defined, yet. It is obvious that the majority of our patients with JE experienced concomitant EGJOO. This could be caused by high fat and carbohydrate intake or even an increased intraabdominal pressure [27]. From a pathophysiological point of view, EGJOO may be responsible for the development of JE in our cohort, as overfilling of laparoscopic adjustable gastric bands, simulating an outflow obstruction, was observed to induce repetitive hypercontractile esophageal contractions in a study of 20 patients [28]. This is in line with the finding that EGJOO was noted in 17.5% of our population, which is significantly more frequent when compared with a prevalence of 4% that was observed in a trial including non-obese [29]. However, only longitudinal trials will reveal if alarm symptoms like dysphagia or non-cardiac chest pain will develop over time or are just masked by an increased sensory threshold analogous to GERD. Hypothetically, reduction of intraabdominal pressure accomplished by successful metabolic surgery may also lead to normalization via a reduction of the intraabdominal pressure. As far as esophageal motility disorders are concerned, asymptomatic patients with morbid obesity seem to be associated with a higher prevalence when compared with non-obese controls. Nevertheless, the clinical significance remains unclear.

Due to the retrospective nature of the study design, potential selection bias cannot be ruled out. However, we compensated this limitation by consecutively including patients, prospective data collection, and objective evaluation.

## Conclusion

This is the first study using a modern approach to diagnose GERD in asymptomatic patients with morbid obesity prior to primary metabolic surgery. We revealed that GERD was common even in asymptomatic individuals with morbid obesity. Esophageal motility disorders were found in 35.6% with the current need of longitudinal trials to delineate therapeutic implications.
